# Métis women’s health and wellbeing scoping review: resurgence in action

**DOI:** 10.1177/11771801251363444

**Published:** 2025-08-11

**Authors:** Janice Cindy Gaudet, Hannah Bouvier, Paulette Dahlseide

**Affiliations:** 1 Faculty Saint-Jean Edmonton, University of Alberta, Canada; 2 College of Law, University of Saskatchewan, Canada; 3 Wapanachkos, Faculty of Medicine & Dentistry, University of Alberta, Canada

**Keywords:** health, Indigenous health, Métis, North America, scoping review, wellbeing, women

## Abstract

Métis (one of three constitutionally recognized Indigenous Peoples in Canada) women’s health and wellbeing research that centers resurgence, resistance, kinship, land, and place offers an intersectional perspective that interrupts deficit-based health narratives. To understand these intersections and emerging pathways, we conducted a Métis women’s health and wellbeing scoping review to get an overall picture of breadth and depth of published articles since 2010. 29 articles fit our scoping criteria. Our findings show that Métis women’s health and wellbeing research offers insight into what Métis women are saying about their own health and wellbeing. They also demonstrate the pivots to broader understanding of identity and gender beyond binary norms, to engagement with two national inquiries, partnerships with political organizations, and COVID-19. The growing uptake on positionality reflects the ways in which Métis-centered epistemologies, methodologies, theories, and methods intersect with Métis women’s health and wellbeing.

## Introduction

Métis (one of three constitutionally recognized Indigenous Peoples in Canada) women have and continue to contribute to the social, political, spiritual, intellectual, and cultural life of what we now call Canada. The health and wellbeing of women has historically been, and continues to be, paramount to the governance and the vitality of our bodies, households, lands, and life itself. The health and wellbeing of Métis women matter as we carry distinct governing and leadership roles and responsibilities for the health and wellbeing of our families, communities, and therefore our Nation broadly. Historical research demonstrates that Métis women’s kinship systems were integral to the wellbeing of Métis Family and Community (Anderson, 2011, 2016; Macdougall, 2017, 2018; Payment, 2001, 2009; Teillet, 2019; Troupe, 2009). We purposefully capitalize Family and Community as it is tied to cultural identity. This is indicative in Métis women’s health and wellbeing research as it gains momentum with the increase of Métis women’s scholarship. In this article, we share the findings of a scoping review on Métis women’s health and wellbeing research. While a Métis health scoping review by Gmitroski et al. (2023) was recently published, our review is specific to Métis women’s health and wellbeing, and was conducted by Métis women. Our analysis is contextualized as resurgence in action and in our respective positionality and privilege to do this work.

Métis scholar Dr. Aubrey Hanson (2020) explores the correlation of cultural and political resurgence in relation to other Indigenous literature. She helps us understand resurgence as “occur[ing] through concrete, everyday existence of individuals and communities demonstrating the continuity of Indigenous lifeways . . . with processes of resurgence inspired by and emerged from Indigenous words, ways and wisdom both old and new” (Hanson, 2020, p. 23). When asking our Michif (Métis identity and language) relative Graham Andrews about how we would consider resurgence in Michif, he shared the Michif meaning of the word, ni-paw-weh (life in motion). He said in the Michif language, “we need to think about it from the perspective of ni-paw-weh and pi-mah-chi-win—we are keeping on, we are in motion, in other words our culture, our pi-ma-chi-win [life] never ceased to exist. Métis life is moving” (G. Andrews, personal communication, 15 July 2024). Deeply touched by the embodied Michif expression of resurgence, we entitle this work resurgence in action. We have chosen to be transparent about the thinking that influences our research.

## Positioning ourselves

We briefly share our positionality as it is important for Métis scholars to include the inspiration, the roots of cultural continuity, and what Dr. Kim Anderson (2016) describes as foundations of resistances. We acknowledge that we also have a responsibility to resist and reject the pervasive negative mentalities that bring us and keep us down. As Métis women, it is imperative to our health and wellbeing to go beyond Western notions of identity and health that perpetuate the objectification of our lives, our bodies, our kinship relations and the wisdom and knowledge that exist within these systems and our stories.

As a Métis health scholar with deep kinship connections to the Métis Communities situated along the South Saskatchewan river also known as the Batoche and St. Laurent Homeland region, I, Janice Cindy Gaudet, am immensely grateful for my continued relationship to these place and peoples. It enriches my understanding of cultural identity, knowledge, and teachings as they relate to kinship health and wellbeing. It is the breath that persists within and through me, challenging me as I explore Métis women’s health and wellbeing in relation to Métis kinship health research methodologies.

I, Hannah Bouvier, grew up learning with and from the medicines of the land. Being raised in Southern Alberta, my kinship ties remain connected in Western Manitoba. From berry picking to dandelions and rose hips, harvesting together was part of our life. It was in recent years that I began to connect more deeply as to how this was tied to my Métis identity, to how my mother and aunties carried so much plant knowledge and have generously shared it with me. In reflecting on this now, I realize that my own knowledge of health and wellbeing is limited without their perspective and knowledge.

I, Paulette Dalseide, am a daughter, auntie, wife, mother of six, grandmother, health professional, and scholar from rural Northeastern Alberta. I have always loved the way we, as Métis people, visit and how this has shaped my understanding of health and community. My parents would take us on visits and warmly welcome guests who came to work, play, or pray. These gatherings were always filled with delicious food and hearty laughter, especially when the aunties were together. The ethic of care inherent in a good visit was mutual and sincere. Growing up with a strong sense of community, I have dedicated much of my 30-year career as a dental hygienist and now health researcher to making oral health care accessible to everyone. To me, my clinical practice, research, and everyday life are inextricably braided together and the values of kinship and care I learn from my family and community are woven into everything I do.

## Building on Indigenous women’s health research

This scoping review builds on the findings of the broader Indigenous women’s health and wellbeing scoping review by Indigenous health scholars McGuire-Adams et al. (2023). These findings demonstrate that theoretical and methodological approaches in Indigenous women’s health and wellbeing influence the stories we tell about Indigenous women’s health and wellbeing as well as the processes and outcomes of research (Loukes et al., 2022; McGuire-Adams et al., 2023). The findings indicate that in the last decade, “while a range of critical theoretical and methodological approaches are increasingly being applied, the use of cultural resurgence and Indigenous feminism in health research is not widespread” (McGuire-Adams et al., 2023, p. 1). Building on these foundational findings and the importance of the complementary role of distinction-based research, our scoping review contributes to Métis-women-specific health and wellbeing research.

Our process drew on the iterative six-step framework for conducting scoping reviews that summarizes and maps existing research topics and gaps across diverse fields of study (Arksey & O’Malley, 2005; Colquhoun et al., 2014). We adapted this process to include a Métis methodology of knowledge exchange through visiting in step 6. The process is as follows: (a) determine the study objectives-goals; (b) identify relevant studies through a database and keyword strategy developed in consultation with a University of Alberta librarian; (c) study the selection based on inclusion and exclusion criteria; (d) chart the data; (e) synthesize the data: collate and describe findings; (f) collaborate with Métis community-engaged researchers, community members, and leaders to inform and validate the scoping review findings (Colquhoun et al., 2014; Lingard & Colquhoun, 2022).

## Our research objectives and goals

We conducted a scoping review to (a) get an overall picture of breadth and depth of existing literature about Métis women’s health and wellbeing and (b) identify and aggregate the research topics informing the field of study. The findings demonstrate a deeper understanding of how Métis women’s epistemologies, methodologies, and methods intersect with Métis perspectives on kinship health and wellbeing. Challenging deficit-based narratives, processes, and analyses is crucial for advancing Métis principles of relationality as experienced and articulated by Métis women themselves. There is a lack of Métis-women-specific health research, and “Métis health data are often integrated with other Aboriginal health data, resulting in a dearth of Métis-specific health research, information, and programming” (Wesche, 2013, p. 188). The uptake of Métis-women-led research offers the possibilities of grounding research in deep living realities, places, identities, and spiritual, social, and political contexts (moving beyond generalities and deficit). This offers opportunities for Métis women to tell their story and to inquire more deeply as to what Métis women’s resurgence in health looks like. How are Métis women leading these efforts, and what tools help us assess our health and wellbeing and the place of culturally relevant practices? Why is this significant to our health and wellbeing? Such inquiries invite new considerations to broaden fields of study.

## Data sources and search

Our inquiry aimed to capture the range and diversity of interdisciplinary research into Métis women’s health and wellbeing. A University of Alberta librarian provided guidance on the basic approaches to database searches and to conducting searches of key terms. As a group, we determined key word search strategies representative of Métis women’s health or wellbeing, policy, and North America in the following databases: Academic Search Complete, Native Health Database, Web of Science, Google Scholar, Bibliography of Native North America, Gender Studies, Indigenous Studies Portal, and JSTOR. Limiting the searches to January 2010 to May 2022, followed by a second search from May 2022 to May 2024, keyword combinations of Métis, women, health and wellbeing, Turtle Island, currently known as Canada, and policy guided our Boolean or database-specific searches (Table 1).

**Table 1.** Search terms used.

Concept	Keywords used
Métis	Métis, Halfbreed, Michif, Métisse, Road Allowance People, Flower Beadwork People, Otipemisiwak. These are the diverse cultural terms that Métis Peoples are referred to or refer to themselves historically and contemporarily.
Women	woman, women, girl, female, femme, faam, gender
Health	health, healthy, wellbeing, well being, well-being, quality of life, wellness, illness
Turtle Island	North America, Canada, United States, Turtle Island
Policy	policy, policies

Métis = one of three constitutionally recognized Indigenous Peoples in Canada; Michif = Métis identity and language; Métisse = mixed; Optipemisiwak = people who own themselves.

## Inclusion and exclusion criteria

We determined the inclusion and exclusion criteria adopted for the scoping review through consensus, choosing to include peer-reviewed articles with a focus on Métis women and their health and wellbeing.

### Inclusion

Some of the researchers blended their results on Métis women with First Nations women, while some differentiated their findings regarding the participants or the results. It was important to consider both. We are also mindful of not contributing to colonial practices that break up Métis and First Nation kin. While understanding the complexity of identity and intermarriages, we included these research studies, given that some of the articles include knowledge holders who hold kinship identities. Our five criteria are as follows:

- research that focuses on Métis women’s health and wellbeing
- research that includes Métis women’s health and wellbeing within a broader family context
- research that includes both Métis and First Nations women that either differentiates or blends the research results
- research that differentiates Métis women and men in research results
- research located on Turtle Island known as North America—Canada and the USA.

### Exclusion

We chose to exclude book chapters, gray literature, and community-centered reports on Métis women’s health and wellbeing, although we acknowledge that these do exist, are often community-led, and are equally important as peer-reviewed scientific articles. Broadly related scoping reviews were also excluded.

- scoping reviews
- research out of date scope
- duplicates
- research specific to Métis men
- research broadly speaking of Métis health through a pan-Indigenous lens meaning First Nations, Métis and Inuit (an Indigenous people of Arctic North America) health research
- book chapters
- research located outside of North America
- Master’s or PhD theses, gray literature
- research on Métis people in general
- other non-journal resources or inaccessible resources

## Data charting

We used the document management tool Zotero to assemble our results and distribute the further tasks of abstract screening and full-text review. A Google Sheet was created to chart eligible articles identifying the following: author, title, abstract, journal, link, year, country, location, participants, Indigenous authorship, discipline, topic of study, research question, methodology, theoretical lens, methods, findings, and type of analysis. To further assess source eligibility, these articles were divided among the team members, and team member concerns regarding eligibility and ineligibility were discussed and determined collectively after two full-text reviews. Eligible dates were determined by online publication date reflecting when the database upholds the files. Detailed charts informed our rigorous process of mapping, cross-referencing, and thematic intersections between articles (Colquhoun et al., 2014).

## Data synthesis: constructing the picture

With detailed spreadsheets and ongoing dialogue to synthesize our data, the construction of the picture was emergent and informed by our scoping review findings. This informs our understanding and interpretation through both ontological and epistemological lenses as part of a dynamic praxis of being in relation. Our theoretical framework is deeply rooted in our positionality and our roles and responsibilities as Métis women in family and community. Throughout our analysis we consider principles of resurgence to guide our collaborative work.

Our aim is to render visible Métis women’s health and wellbeing research from 2010 to 2024. We consider what patterns and trends are emerging and, what the overall landscape reveals about the field of Métis women’s health and wellbeing research. We ask: Where do we invest our pi-ma-chi-win moving forward, in a way that benefits our health and the health and wellbeing of our families, communities, and homelands? What topics are being studied or developed? How has the picture changed from 2010 to 2024, and where is the field going? In conclusion, we reflect on how hopeful this picture is.

## Engaging with our peers and community

Engagement in the various stages of the scoping review process offers opportunities for community “to suggest additional references and provide insights beyond those in the literature” (Colquhoun et al., 2014, p. 1294). In July 2023, we invited our Métis colleagues to a research picnic during the annual Back to Batoche Days, an important cultural festival on the Métis Batoche Homeland. We visited, listening to each of our peers sharing their family connections, their positions, research, and interests. Despite our diverse backgrounds and unique social and historical contexts, we all came together in a dedicated visiting tent, where we shared meals and discussions about our scoping review process, preliminary findings, and research interests. These interactions remain ongoing, as does the storied approach (Lingard & Colquhoun, 2022).

## Results

### Data selection

**Figure 1.**
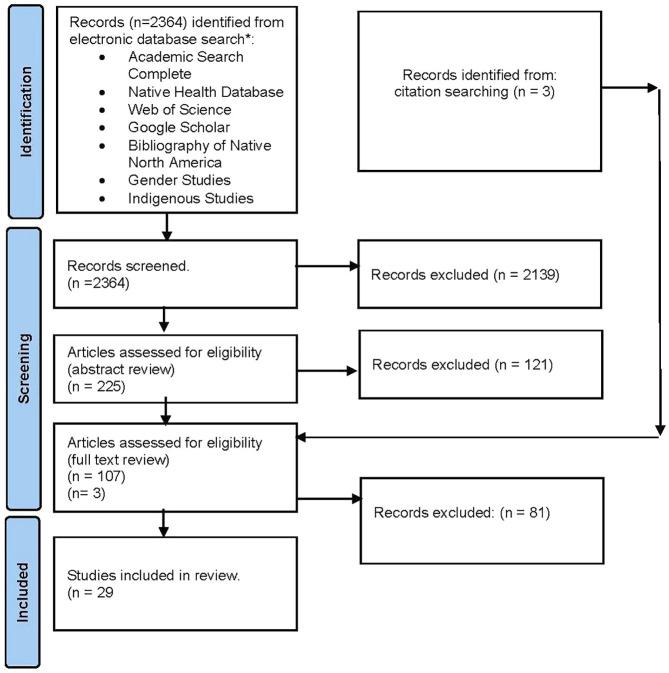
Data selection.

The initial database search in May 2022 yielded 2,096 results, with a second search, May 2022–May 2024, yielding 267 results for a total of 2,364 articles. After duplicates were removed, reviewers scanned titles and keywords as per the eligibility criteria, resulting in 225 remaining sources of peer-reviewed articles, book chapters, and other resources that met search requirements. Policy and North America were not often met in the keyword search. The 225 sources were then transferred and organized into Zotero. After review, 121 articles were excluded—duplicates, book chapters, and other non-journal resources. The remaining 107 articles were then sorted on Google Sheets to allow for further analysis and to determine continued eligibility within the 14-year period from 2010–2024. A hand search of the reference list of the previously noted broader Indigenous women’s health scoping review (McGuire-Adams et al., 2023) yielded three additional sources that were added for final consideration and analysis. After completing a full-text analysis of these 110 articles, we found 29 articles that met our study criteria (Figure 1).

### Findings

**Figure 2.**
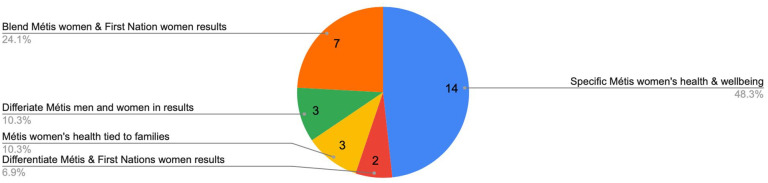
Eligibility criteria. Métis = one of three constitutionally recognized Indigenous Peoples in Canada.

Our eligibility criteria retrieved 29 peer-reviewed journal articles to which we discuss our findings throughout the next following sections. The chart below demonstrates that 14 (48.3%) research articles are specific to Métis women’s health and wellbeing, three (10.3%) include both Métis and First Nations women and differentiate the findings, seven (24.1%) blend Métis women and First Nations women in research results, three (10.3%) consider Métis women’s health as tied to family wellness, and three (10.3%) include Métis men and women and differentiate the results. The literature explores an array of topics including Métis women’s biomedical and holistic health needs, access to care, the challenges of navigating oppressive health service systems, and the exclusion and inclusion of Métis women’s voices and perspectives. It also examines collective solutions to improve culturally safe service provision. There is an emerging focus on Métis women’s research methodologies, methods, and identity within health and wellbeing study. Fourteen of the 29 articles (48.2%) were specific to Métis women’s health and wellbeing. Eleven of those 14 (78.6%) were led by Métis women scholars (Figure 2).

### The growth of Métis women’s health and wellbeing research

*Overview: 2010–2019*: Our review shows that there is growing academic attention placed on Métis women’s health and wellbeing. The earliest article, authored by Kumar et al. (2012), includes Métis women’s health in a comparison of suicide ideation between Métis men and women appearing in 2012, with increase in publication over the next 7 years to 13 research articles by the end of 2019. Out of these 13, five are Métis-women’s-health-specific (Krieg & Martz, 2013; LaVallee et al., 2016; Monchalin & Monchalin, 2018; Monchalin et al., 2019; Wesche, 2013), and three out of 13 include and differentiate Métis women’s and men’s health (Foulds et al., 2013; Jandoc et al., 2015; Kumar et al., 2012). Métis men are no longer included in research studies after 2015. Métis woman Elders appear only in 2013 (Krieg & Martz, 2013). Five of the 13 blend Métis and First Nations women’s health research results (Cooper & Driedger, 2019; Cooper et al., 2019a, 2019b; Ferguson et al., 2018; Lawrence et al., 2016). The first place-based literature specific to Métis Elders is conducted in a Métis-specific context of Buffalo Narrows, Saskatchewan (Krieg & Martz, 2013). We note a growth of place-based research whereby research is situated within a particular geographical location that holds unique histories, knowledges and lived experiences and therefore cultural, social and ontological context in 2019. This shifts from more generalized research and points to the influence of positionality and place-based research on choices of theories, methodologies, and methods.

In 2016, our scoping review identified a notable foundational article developed by three Métis scholars (LaVallee et al., 2016). It is foundational as it points to critical self-reflection, the importance of relationality among researchers, and the invaluable guidance of female Elders as essential components of research methodology. The authors draw from a research project that identified the prevalence of racism, discrimination, poverty, lower levels of education, over-crowding, lower water quality, and food insecurity” in relation to tuberculosis in Métis communities (LaVallee et al., 2016, p. 169). With respect to their Métis research ethics and protocol, they explain: “In many ways, we recognize that we are relearning Métis knowledge for ourselves, so that we can share it with the next generation. This is one of the ways that we give meaning to our wellness” (LaVallee et al., 2016, p. 162).

In 2018, another foundational article by Renée Monchalin and Lisa Monchalin (2018) on Métis women calls to action the importance of listening to Métis women, given their role in their respective community and family health. This knowing directs their future research and problematizes the “silencing of female narratives” (Monchalin & Monchalin, 2018, p. 19) rooted in colonial legislation, patriarchal influence, gender binaries, and land displacement. They present the lack of Métis-specific health data, which creates invisibility in “provincial and territorial health data sets” (Monchalin & Monchalin, 2018, p. 20). This is the beginning of research that critiques pan-Indigenous “health research” and recognizes the “lack of culturally-specific health services” (Monchalin & Monchalin, 2018, p. 20). Their research is positioned as building on the central role of Métis women in shaping, retaining, maintaining, and persistence of “Métis identity” (Monchalin & Monchalin, 2018, p. 21). They remind their readers that “we do not need saving” (Monchalin & Monchalin, 2018, p. 23). They provide a crucial perspective that shifts away from victimizing, deficit-focused, and trauma-centered research, challenging behavior-oriented studies and questioning complicity with patriarchal norms. This is the beginning of research that builds on our Métis health colleuges longitudinal Métis women’s health research project called Our Health Counts (Auger et al., 2022; Jones et al., 2024; Monchalin & Monchalin, 2018; Monchalin et al., 2019; Monchalin, Smylie, & Bourgeois, 2020; Monchalin, Smylie, & Nowgesic, 2020).

**Figure 3.**
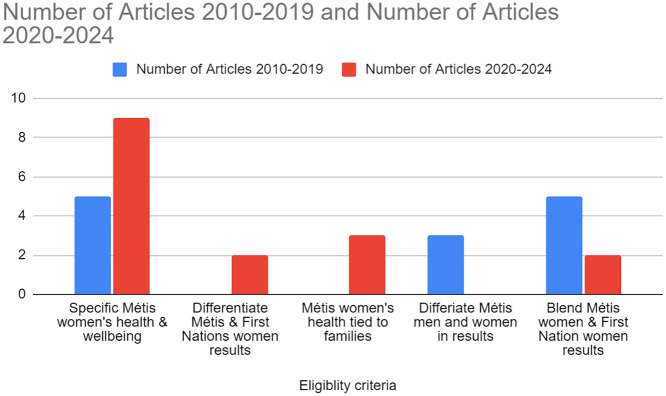
Eligibility criteria. Métis = one of three constitutionally recognized Indigenous Peoples in Canada.

*Overview: 2020 to 2024*: Our review demonstrates a notable uptake of Métis women’s health and wellbeing research from the period of 2020–2024 (Figure 3). In this 4-year span, 16 research articles met our criteria, signaling a significant increase in both the total number of articles and the rate of publication in this field of study. During this window, place-based research, signaling the distinction of urban, rural, and Métis communities and local realities, are considered within two broad quantitative studies on maternal health (Struck et al., 2021; Volklander et al., 2024). These were conducted in collaboration with Métis political organizations. We note a slight increase in research that is Métis-women-specific in this period, and a decrease in research that blends Métis and First Nations women. From 2021 to 2024, four of the 16 research articles follow a blended cohort of Métis and First Nation women, with half of these distinguishing between the two groups in the results, departing from the pan-Indigenous focus of 2012–2019. Of the articles distinguishing Métis data and results, one examines access and equity challenges among Indigenous women, discussing their children’s experiences of oral health (Kyoon-Achan et al., 2021), and the other looks at the disparities of emergency health care among off-reserve Indigenous women (Srugo et al., 2023). Of the two other articles that blend the data, one is a quantitative study on diabetic retinopathy eye care (Umaefulam & Premkumar, 2023), while the other is a best practice reflection on Indigenous knowledge exchange (Cooper & Driedger, 2021). Through our review, we identified three articles that connect Métis women’s wellbeing to family (Auger, 2021; Jones et al., 2020; Struck et al., 2021) pointing to ways that wellness is deeply interconnected to kinship and is not age-specific. Linking new generations to Elders significantly enhances Métis women’s wellbeing. Struck et al. (2021) highlight positive birth outcomes for Métis women as crucial for their overall wellbeing and that of their communities. Quantitative research underscores the necessity of prenatal care and diabetes management to improve birth outcomes and to reduce socioeconomic inequity (Voaklander et al., 2023). Jones et al. (2020) emphasize the vital role Métis women play in cultural knowledge transmission with intergenerational teachings informed by kinship promoting wellness across generations, and the impact these connections can have to support multi-generation wellness. Auger et al. (2022) stress that engaging families in knowledge translation efforts is essential for ensuring the wellness and wellbeing of future generations. These articles collectively underscore the critical role of family connections and intergenerational teachings in enhancing the overall wellness of Métis women.

### Kinship, place, and positionality

Our findings highlight a pivot in the field with a notable increase in research authored and led collaboratively by Métis scholars (Auger, 2021; Auger et al., 2022; Flaminio et al., 2020; Gaudet et al., 2020; Jones et al., 2020, 2024; Monchalin et al., 2019, 2024; Monchalin, Smylie, & Bourgeois, 2020; Monchalin, Smylie, & Nowgesic, 2020; Paul et al., 2023). This is reflected in the article titles, such as *I Would Prefer to Have My Health Care Provided over a Cup of Tea Any Day* (Monchalin et al., 2019). In addition to ongoing research led and co-led by Monchalin, nine research papers are authored and led by Métis women scholars positioning their research in relation to place, kinship, and community. Also demonstrated in these works is research rationale that shows that “Métis women held knowledge central to the health and well-being of their communities” (Monchalin et al., 2019, p. 217). The philosophical principle and purpose was to learn with and from one another as Métis women’s kinship thereby upholding woman’s health and wellbeing in Family and Community governance. This knowing is demonstrated the relational, ethical and methodological approaches amplified in one of our findings (Auger, 2021; Auger et al., 2022; Flaminio et al., 2020; Gaudet et al., 2020; Monchalin et al., 2019, 2024).

Two articles are Métis-women-led in relation to a specific geographical location within the broader Batoche Homeland and with Métis women with whom the researchers have kinship ties (Flaminio et al., 2020; Gaudet et al., 2020). This literature contributes to relational approaches to community-centered research. It speaks of decoloniality as a return “to their own communities, to their own Métis women’s stories, and epistemological grounding in learning from our Aunties” (Gaudet et al., 2020, p. 13). There is a recognition that Métis communities are not homogeneous. We are building on research that unsettles the colonial myth of sameness, and therefore the myth of same solutions for all Métis women’s health and wellbeing. A move toward equitable and place-based research disrupts one-size-fits-all research approaches.

### Urban and rural distinctions

Urban-centered Métis health and wellbeing research challenges the settler-colonial erasure of Métis folks in urban spaces and addresses a misconception of “Canadian cities as non-Indigenous” (Auger et al., 2022, p. 22). These Métis scholars invite thinking from their respective “self-location on decolonizing and thinking around identity,” and “assert the need to return to our teachings, which have long been upheld by powerful knowledge keepers, Elders and Matriarchs across Métis community” (Auger et al., 2022, p. 34). Auger’s (2021) decolonial research is deeply connected to her familial ways of knowing, sense of nationhood, and her commitment to Métis youth. She calls for more Métis-youth-centered research and is part of another research project that signals in their title kinship-ways of being and moving in between spaces and places “It’s not like I’m more Indigenous there, and less Indigenous-here” (Monchalin, Smiley, & Bourgeois, 2020, p. 323).

Three other urban-centered research projects in 2019 and 2020 are Métis-women-led and focus on Métis women’s understanding of health and wellbeing as connected to culture and identity. These studies are connected to a broader longitudinal Our Health Counts project, and the Seventh Generation Midwives in Toronto. Decolonial and Indigenous feminist theories are applied presenting a critique of patriarchal and political narratives that have “silenced Métis women for generations” (Monchalin, Smylie, & Bourgeois, 2020, p. 325). Their study expands to Métis women, trans, or other, which we discuss further in our analysis on gender binaries.

Our findings show that cultural continuity is part of Métis women’s health and wellbeing research methodologies. Métis scholar Auger locates herself in a grounded theoretical approach, making this correlation with her kin in British Columbia. “I introduce myself and my relations to my community and this research” (Auger, 2021, p. 73). As she points out, strengths-based approaches are framed within the context of the “individual and collective strengths of our families and communities” (Auger, 2021, p. 73). She presents an important critique of the concept of strengths-based research. She highlights the ways in which a theoretical worldview informs the use of the term strength. One is lived and embodied; one is observed from the outside. This is an important consideration given the studies that draw on “strength-based” frameworks with or on urban First Nations and Métis women and girls (Cooper & Dreidger, 2021; Cooper et al., 2019a, 2019b). The tensions and complexity of positionality and cultural identity in the uptake of community-based participatory research lack critical Indigenous and feminist theoretical analysis and risk reproducing deficit-based research (McGuire-Adams et al., 2023).

Another urban study with the Saskatoon Friendship Center and Saskatchewan Health Authority (Umaefulam & Premkumar, 2023) points to the need to increase knowledge about and access to provincial health insurance coverage. In this study, out of the 35 First Nations and Métis women, nine were Métis and “living with or at risk of diabetes” (Umaefulam & Premkumar, 2023, p. 4). Discussions engaged in an important critique on the resilience of the Métis and First Nations women as having agency of their own health and the “prejudice and low empathy and concern for Indigenous women opinions and feelings” (Umaefulam & Premkumar, 2023, p. 7). This is framed as a barrier to proper diabetes eye care. It is the only study that applies self-determination theory to complete the analysis while implying validity given there was no pre-existing relationship between researcher and researched.

Another community-engaged rural study examined Métis Buffalo Narrows local community health with a research team in and with the community (Krieg & Martz, 2013). This is the only article that engages with northern Métis communities and focuses on Métis elderly women’s health and wellbeing needs. The authors demonstrate the challenges facing northern communities when accessing health-care services and the impact of this situation on elderly Métis women’s wellbeing.

### Métis women’s research ethics and Indigenous feminist theory

Notably, three articles apply an Indigenous feminist and decolonial theoretical lens (Monchalin et al., 2019; Monchalin, Smylie, & Bourgeois, 2020; Monchalin, Smylie, & Nowgesic, 2020). The conscious engagement of Indigenous feminist theory and praxis reveal a noticeable shift in the discourse which jumps out in their paper titles. Discourse, the ways in which stories are told, and what stories are told are all informed by the theoretical lens, which points to the importance of Indigenous feminist theory, and its rarity in Métis women’s health and wellbeing research. Research led by Métis women centers what Métis women know they need, thereby affirming Métis women’s stories, embodiment, and knowledge as Métis kinship theory in praxis.

Findings demonstrate that Métis-women-led research engages with cultural and relational ethics and gifting protocols. The cultural practices of gifting, feasting, and togetherness clearly reflect a deep generosity and respect. Engaging with Elders, working with women one is in relation with, becomes another form of enacting health and wellbeing (LaVallee et al., 2016). The spirit of gifting in research also includes local Métis artists, traditional beadwork, berries from the land, sourcing local communities to utilize spaces and to prepare meals, eating together, and honorariums as part of relational ethics, respectable research, and cultural continuity in research methods (Auger et al., 2022; Flaminio et al., 2020; Gaudet et al., 2020; LaVallee et al., 2016; Monchalin, Smylie, & Bourgeois, 2020; Monchalin, Smylie, & Nowgesic, 2020; Paul et al., 2023).

### Culturally safe and unsafe

The literature advocates for access to culturally safe care (Jones et al., 2024; Monchalin et al., 2022; Monchalin & Monchalin, 2018; Paul et al., 2023). In many instances, researchers consciously create cultural safety through their research methods. While cultural safety is not explicitly defined, it is proposed by the Métis women themselves, demonstrating the significance of Indigenous feminist theory in praxis (Monchalin et al., 2019; Monchalin, Smylie, & Bourgeois, 2020; Monchalin, Smylie, & Nowgesic, 2020). The research centers the ways in which Métis women navigate health-care systems, as part of Métis women’s agency. This includes Métis women’s refusal of the historical and ongoing deficit narrative of “blaming Métis communities for their own health problems, more specifically tuberculosis and other diseases” (Jones et al., 2020, p. 118).

Our findings reveal more urban-specific challenges that Métis women experience in terms of access to health care, culturally unsafe or discriminatory care, racism, and sexism, than rural accounts which point to limited health-care access and services. The literature also highlights recommendations from Métis women to address these challenges. Studies point to complexity in identity and how identity is tied to Métis women’s health. Scoping review findings led by Métis women scholars highlight the cultural kinship roles and responsibilities of Métis women when it comes to health care.

This speaks to cultural continuity and respect for the leadership Métis women would have always provided. In consciously pivoting to needs-based research, choice of language matters, given that words are shaped by ideologies. Research conducted by Monchalin and colleagues centers Métis women’s health and wellbeing because they “carry practical solutions for improving access to health and social services,” which includes non-judgment, trauma-informed work, and being “willing to listen and . . . believe Métis women and not minimize their experience” (Monchalin et al., 2022, p. 332). In our visiting, we reflected on Métis-women-led research creates and advocates for solutions and recommendations for and by Métis women.

### Métis women’s identity and the impacts on their health and wellbeing

Identity tensions are linked to funding discrepancies between First Nations Peoples and Métis Peoples, colonial-influenced identities, blurred jurisdictional responsibilities, along with discriminatory care and limited access due to race-based identity, gender, and White passing. Understanding identity is integral given that it is a social determinant of Métis women’s health and wellbeing. This points again to the importance of community-specific knowledges and building in “accountability” in cultural knowledge and safety trainings (Monchalin et al., 2022, p. 330).

Several articles discuss the problems of identity politics and structural racism in relation to jurisdictional responsibilities impacting access to funding, insurance, clinics, and optimum health-care services (Jones et al., 2024; Kyoon-Achan et al., 2021; Lawrence et al., 2016; Paul et al., 2023; Monchalin, Smylie, & Burgeois, 2019; Srugo et al., 2023). Racism is recognized in the literature as “a distal social determinant of health among Indigenous peoples” (Lawrence et al., 2016, p. 180). The findings note the lack of Métis-related services in comparison to First Nations services. In the literature, we learn about the tensions for White passing or presenting Métis and their privilege and challenges (Monchalin, Smylie, & Bourgeois, 2020; Paul et al., 2023). White passing meaning visibly presenting as White allows women to only disclose their identity when they feel safe and compelled to do so (Paul et al., 2023). These tensions are deeply entrenched, informed and maintained by colonial policies, identity politics, and attitudes that divided kinship systems, thereby widening the gap in health services for Métis peoples and contributing to a lack of Métis-specific data (Monchalin & Monchalin, 2018).

Auger et al. (2022) draw on the wisdom of Métis Elders when addressing the tensions of identity politics. They problematize this issue rooted in “patriarchal notions and court decisions on who does not belong” (Auger et al., 2022, p. 34). They also highlight the ongoing impacts of colonialism and systems of oppression which result in lateral violence, and racialized othering as an ongoing form of colonial violence. They demonstrate how Métis women’s health and wellbeing is strengthened through their respective wisdom, connection, and responsibility to the land and children, alongside the place of food from the land, ceremony, connection to family and community, artistic expression of identity, and visiting.

Research within the urban context offers insights into identity that differ from Métis-community-specific research where everyone knows one another. Métis women develop strategies to navigate the complexity of Métis identity within an urban context. For instance, some Métis women refuse and or “actively avoid to disclose their identity out of fear of experiencing racial discrimination, policing, outright denial of medication, and child apprehension” (Paul et al., 2023, p. 255). Métis women share that they experience condescending attitudes when it comes to their health and wellbeing, and medical interventions that do not serve their best interest.

### Beyond gender binaries

The findings show that more recent research has expanded gender terminology beyond the traditional binary of women and men to include two-spirit, trans, and diverse gender identities. Three Métis-women-led research studies offer a deepened understanding to deconstruct Western notions of gender (Auger et al., 2022; Jones et al., 2024; Monchalin et al., 2022). The researchers, engaged in Indigenous critical theory, reflect on their positionality and what those intersections meant for the research.

As we reflect on the knowledge shared throughout these conversations, we are reminded how Métis women, Two-Spirit and gender diverse peoples’ stories are medicine; and we, as a research team, felt honored and uplifted through the process of working through these stories and knowledges and sharing them in the context of Métis identity. (Auger et al., 2022)

Two-spirit identity challenges traditional binary gender roles, signaling that hetero-patriarchal frameworks must be acknowledged and critiqued in Métis women’s health and wellbeing research. Another quantitative study further challenges the gender binary by identifying research participants as “Métis pregnant people” (Voaklander et al., 2023, p. 1533). This research is actively countering the hetero-patriarchal assumption that every person who has a womb identifies as a woman.

### Two national inquiries and United Nations Declaration on the Rights of Indigenous Peoples

Our findings show that in 2018, research begins to reference two recent national inquiries: the Truth and Reconciliation Commission of Canada (2015) inquiry and the National Inquiry into Missing and Murdered Indigenous Women and Girls (2019). Our findings note four research studies (Cooper & Driedger, 2019; Jones et al., 2020; Monchalin et al., 2022; Srugo et al., 2023) that mention one or both of these inquiries. One article that addresses prenatal and maternal care mentions United Nations Declaration on the Rights of Indigenous Peoples (UNDRIP; Struck et al., 2021).

### Pandemic

By 2020, our findings begin to see the place of COVID-19 in Métis women’s health and wellbeing research: the impacts, adaptations, inequities, and violence, and the importance of youth leadership. Five articles discuss the global pandemic (Jones et al., 2020, 2024; Monchalin et al., 2022; Paul et al., 2023; Srugo et al., 2023). Some research continued during COVID-19, demonstrating the importance of adapting and supporting kinship through research, while some highlighted inequities revealed by COVID-19, and its disproportionate impact on Métis women. For instance, the literature demonstrates that public health “stay at home” measures “resulted in a sharp rise of violence against Métis Women and First Nation girls and women” (Jones et al., 2020, p. 120). Only one study recognizes violence within this context in relation to ongoing oppressive systems.

### Research and political partnerships

Our findings illustrate that Métis political entities are consistently involved in partnering with researchers. Out of the 29 research studies, 10 (34%) partnered with Métis political bodies across the homeland. Four out of the 10 are quantitative studies (Jandoc et al., 2015; Kumar et al., 2012; Struck et al., 2021; Volklander et al., 2024). These topics of study include (a) a suicidal ideation comparison between Métis men and women; (b) a comparative analysis of osteoporosis management among Métis men and women; (c) policy development specific to prenatal, maternal, and child health-care needs; and (d) a comparative and population-based study on pre-existing gestational diabetes in pregnant Métis people (Volklander et al., 2024). Another independent quantitative study shows that Métis women are more likely to have cardiovascular disease (Foulds et al., 2013).

This speaks to the ways in which Métis political bodies benefit from quantitative research for policy and advocacy purposes, so as to address gaps in federal jurisdictional responsibility, increase funding access, and improve health access and outcomes for Métis peoples. The findings reveal the need for increased policy development when it comes to equitable access to Métis women’s health and wellbeing. This is also evidenced in Métis-women-led research, as discussed earlier, given that Métis women know what they need to improve their health and wellbeing. However, what the literature shows is that quantitative studies do not consider the place of positionality as an integral part of research in the same manner as Métis women’s health and wellbeing research. Funding agencies tied to political bodies require quantitative data results to demonstrate inequities and ensure ongoing funding. However, there are “distinctive complexities” with funding and identity shaped by colonial ideologies, and “overlapping” geographical and kinship understandings of community are contradictory to funding that demands “clear boundaries” (Evans et al., 2012, p. 61).

There were six qualitative studies with political collaborations. One particular qualitative study (Kyoon-Achan et al., 2021) aims to inform early childhood oral health, distrust of health-care professionals, and access and equity in health-care systems. Another (Wesche, 2013) points to the sexual exploitation risks Métis and First Nations women experience in three urban centers. The findings conclude that “system change that addresses the social determinants of health must be fostered through culturally appropriate policy and practice at multiple levels” (Wesche, 2013, p. 187). This is the only study that speaks of sexual exploitation experienced by Métis and First Nations women in health services. Four other qualitative research studies (Cooper & Dreidger, 2019; Cooper et al., 2019a, 2019b) that blend First Nations and Métis women’s perspectives of health and wellbeing in urban contexts are framed with strength-based and ethical approaches to research. We discussed the implications of a strength-based approach that lacks critical theoretical underpinnings of positionality and risk perpetuating deficit-research.

## Concluding thoughts

Findings from this scoping review offer important insights and discussions regarding Métis women’s health and wellbeing research: emerging trends, research topics, culture, identity, kinship, positionality, gender, politics, and leadership. We want to express our gratitude for the opportunity to visit with Métis women scholars and each other, and to learn about the diverse range of health and wellbeing research involving Métis women. The picture of our health and wellbeing is incomplete without Indigenous women themselves engaged in their own health research (Anderson, 2008; McGuire-Adams, 2021). Métis-women-led research informs a clearer picture of ni-pah-weh pimachin (our life in motion) as they share their wisdom, experiences, and practical health and wellbeing needs. For instance, it was hopeful to hear Métis women affirm “I understand my eco-system” (Monchalin et al., 2022, p. 331), and ask “What are the pillars in our lives that do hold us up and lift us up and keep us healthy?” (Monchalin et al., 2022, p. 331). This highlights the practices that sustain our pi-ma-chi-win, such as “visiting and gathering” together (Flaminio et al., 2020). With relationality, cultural resurgence, and critical theories as emerging guideposts in Indigenous health and wellbeing research, ill-health and deficit narratives shift; we turn toward one another, our land, our kin, our stories, and our theories, epistemologies, and methodologies. This movement serves to reorient research approaches to render visible the complexities, the places, and the people that are intimately tied to Métis women’s health and wellbeing, individually and collectively. This is a way to both correct and counter dominant narratives and research that centers health disparities, erasure, loss, illness, trauma, dispossession, and violence.

Indigenous women’s theories, methodologies, and epistemologies remain limited in the scoping review findings. Métis women’s health research cannot exclusively focus on health discrepancies, nor can we deny it exists. Behavior-focused analysis negates broader patriarchal ideologies, the colonial legacy, heteronormativity, and ongoing systemic racism and their impact on Métis women’s health and wellbeing (Hyett et al., 2019). The visibility of a theoretical lens points to the assumption that Métis critical analysis and close community context is understood, and nuanced, in community-engaged research and collaborations with Métis political entities. For these reasons, critical theoretical analysis and clarity of framework are vital to the pimachin (life) we carry and that carry us.

## References

[bibr1-11771801251363444] Anderson K. (2008). Notokwe opikiheet—“Old-lady raised”: Aboriginal women’s reflections on ethics and methodologies in health research. Canadian Woman Studies, 26(3–4), 6–12.

[bibr2-11771801251363444] Anderson K. (2011). Life stages and Native women: Memory, teachings, and story medicine. University of Manitoba Press.

[bibr3-11771801251363444] Anderson K. (2016). Recognition of being: Reconstructing Native womanhood. Women’s Press of Canada.

[bibr4-11771801251363444] Arksey H. O’Malley L. (2005). Scoping studies: Towards a methodological framework. International Journal of Social Research Methodology, 8(1), 19–32. 10.1080/1364557032000119616

[bibr5-11771801251363444] Auger M. (2021). “The strengths of our community and our culture”: Cultural continuity as a determinant of mental health for Métis people in British Columbia. Turtle Island Journal of Indigenous Health, 1(2), 71–83. 10.33137/tijih.v1i2.36046

[bibr6-11771801251363444] Auger M. Jones C. Monchalin R. Paul W. (2022). “It’s in my blood. It’s in my spirit. It’s in my ancestry”: Identity and its impact on wellness for Métis women, two-spirit, and gender diverse people in Victoria, British Columbia. First Peoples Child & Family Review, 17(1), Article 1.

[bibr7-11771801251363444] Colquhoun H. L. Levac D. O’Brien K. K. Straus S. Tricco A. C. Perrier L. Kastner M. Moher D. (2014). Scoping reviews: Time for clarity in definition, methods, and reporting. Journal of Clinical Epidemiology, 67(12), 1291–1294. 10.1016/j.jclinepi.2014.03.013 25034198

[bibr8-11771801251363444] Cooper E. J. Driedger S. M. (2019). “If you fall down, you get back up”: Creating a space for testimony and witnessing by urban Indigenous women and girls. The International Indigenous Policy Journal, 10(1), Article 1. 10.18584/iipj.2019.10.1.1

[bibr9-11771801251363444] Cooper E. J. Driedger S. M. (2021). The importance of explicit and timely knowledge exchange practices stemming from research with Indigenous families. Qualitative Report, 26(8), 2405–2443. 10.46743/2160-3715/2021.3835

[bibr10-11771801251363444] Cooper E. J. Driedger S. M. Lavoie J. G. (2019a). Building on strengths: Collaborative intergenerational health research with urban First Nations and Métis women and girls. International Journal of Indigenous Health, 14(1), 107–187. 10.32799/ijih.v14i1.31932

[bibr11-11771801251363444] Cooper E. J. Driedger S. M. Lavoie J. G. (2019b). Employing a harm-reduction approach between women and girls within Indigenous familial relationships. Culture, Medicine, and Psychiatry, 43(1), 134–159. 10.1007/s11013-018-9603-x 30121724

[bibr12-11771801251363444] Evans M. Andersen C. Dietrich D. Bourassa C. Logan T. Berg L. D. Devolder E. (2012). Funding and ethics in Métis community based researcher: The complications of a contemporary context. International Journal of Critical Indigenous Studies, 5(1), 54–66. 10.5204/ijcis.v5i1.94

[bibr13-11771801251363444] Ferguson L. Epp G. B. Wuttunee K. Dunn M. McHugh T.-L. Humbert M. L. (2018). “It’s more than just performing well in your sport. It’s also about being healthy physically, mentally, emotionally, and spiritually”: Indigenous women athletes’ meanings and experiences of flourishing in sport. Qualitative Research in Sport, Exercise and Health, 11(1), 1–19. 10.1080/2159676X.2018.1458332

[bibr14-11771801251363444] Flaminio A. C. Gaudet J. C. Dorion L. M. (2020). Métis women gathering: Visiting together and voicing wellness for ourselves. AlterNative: An International Journal of Indigenous Peoples, 16(1), 55–63. 10.1177/1177180120903499

[bibr15-11771801251363444] Foulds H. J. A. Shubair M. M. Warburton D. E. R. (2013). A review of the cardiometabolic risk experience among Canadian Métis populations. Canadian Journal of Cardiology, 29(8), 1006–1013. 10.1016/j.cjca.2012.11.029 23465285

[bibr16-11771801251363444] Gaudet J. C. Dorion L. M. Flaminio A. C. (2020). Exploring the effectiveness of Métis women’s research methodology and methods: Promising wellness research practices. First Peoples Child & Family Review, 15(1), Article 1.

[bibr17-11771801251363444] Gmitroski K.-L. Hastings K. G. Legault G. Barbic S. (2023). Métis health in Canada: A scoping review of Métis-specific health literature. CMAJ Open, 11(5), E884–E893. 10.9778/cmajo.20230006 PMC10558240 37788865

[bibr18-11771801251363444] Hanson A. J. (2020). Literatures, communities, and learning: Conversations with Indigenous writers. Wilfred Laurier University Press.

[bibr19-11771801251363444] Hyett S. L. Gabel C. Marjerrison S. Schwartz L. (2019). Deficit-based Indigenous health research and the stereotyping of Indigenous peoples. Canadian Journal of Bioethics, 2(2), 102–109. 10.7202/1065690ar

[bibr20-11771801251363444] Jandoc R. Jembere N. Khan S. Russell S. J. Allard Y. Cadarette S. M. (2015). Osteoporosis management and fractures in the Métis of Ontario, Canada. Archives of Osteoporosis, 10(1), Article 12. 10.1007/s11657-015-0212-9 PMC4412654 25910866

[bibr21-11771801251363444] Jones C. Auger M. D. Paul W. Monchalin R. (2024). “I’m still not over feeling so isolated”: Métis women, two-spirit, and gender-diverse people’s experiences of the COVID-19 pandemic. Canadian Journal of Public Health, 115(2), 199–208. 10.17269/s41997-023-00849-3 38231468 PMC11006636

[bibr22-11771801251363444] Jones C. Monchalin R. Bourgeois C. Smylie J. (2020). Kokums to the Iskwêsisisak: COVID-19 and urban Métis girls and young women. Girlhood Studies, 13(3), 116–132. 10.3167/ghs.2020.130309

[bibr23-11771801251363444] Krieg B. Martz D. (2013). Meeting the health care needs of elderly Métis women in Buffalo Narrows, Saskatchewan. International Journal of Indigenous Health, 4(1), 34–41. 10.18357/ijih41200812313

[bibr24-11771801251363444] Kumar M. B. Walls M. Janz T. Hutchinson P. Turner T. Graham C. (2012). Suicidal ideation among Métis adult men and women–associated risk and protective factors: Findings from a nationally representative survey. International Journal of Circumpolar Health, 71(1), Article 18829. 10.3402/ijch.v71i0.18829 PMC3417687 22901287

[bibr25-11771801251363444] Kyoon-Achan G. Schroth R. J. DeMaré D. Sturym M. Edwards J. M. Sanguins J. Campbell R. Chartrand F. Bertone M. Moffatt M. E. K. (2021). First Nations and Métis peoples’ access and equity challenges with early childhood oral health: A qualitative study. International Journal for Equity in Health, 20(1), Article 134. 10.1186/s12939-021-01476-5 PMC8183050 34098968

[bibr26-11771801251363444] LaVallee A. Troupe C. Turner T. (2016). Negotiating and exploring relationships in Métis community-based research. Engaged Scholar Journal: Community-Engaged Research, Teaching, and Learning, 2(1), 167–182. 10.15402/esj.v2i1.205

[bibr27-11771801251363444] Lawrence H. P. Cidro J. Isaac-Mann S. Peressini S. Maar M. Schroth R. J. Gordon J. N. Hoffman-Goetz L. Broughton J. R. Jamieson L. (2016). Racism and oral health outcomes among pregnant Canadian Aboriginal women. Journal of Health Care for the Poor and Underserved, 27(1), 178–206. 10.1353/hpu.2016.0030 27763440

[bibr28-11771801251363444] Lingard L. Colquhoun H. (2022). The story behind the synthesis: Writing an effective introduction to your scoping review. Perspectives on Medical Education, 11(5), 1–6. 10.1007/s40037-022-00719-7 35960445 PMC9582165

[bibr29-11771801251363444] Loukes K. A. Ferreira C. Gaudet J. C. McGuire-Adams T. (2022). A methodological protocol for conducting a scoping review of health research on/by/with Indigenous women in North America. Systematic Reviews, 11(1), Article 233. 10.1186/s13643-022-02080-6 PMC9615379 36307820

[bibr30-11771801251363444] Macdougall B. (2017). Land, family and identity: Contextualizing Métis health and well-being. National Collaborating Centre for Indigenous Health. https://policycommons.net/artifacts/1888213/land-family-and-identity/2638194/

[bibr31-11771801251363444] Macdougall B. (2018). Knowing who you are: Family history and Aboriginal determinants of health. In Greenwood M. de Leeuw S. Lindsay N. M. (Eds.), Determinants of Indigenous peoples’ health: Beyond the social (2nd ed., pp. 127–146). Canadian Scholars.

[bibr32-11771801251363444] McGuire-Adams T. (2021). “This is what I heard at Naicatchewenin”: Disrupting embodied settler colonialism. Journal of Indigenous Wellbeing, 6, 65–77.

[bibr33-11771801251363444] McGuire-Adams T. Gaudet J. C. Loukes K. A. Ferreira C. (2023). A scoping review of theoretical lenses and methodological approaches in Indigenous women’s health and well-being research in North America over the past two decades. International Journal of Environmental Research and Public Health, 20(8), Article 5479. 10.3390/ijerph20085479 PMC10138645 37107761

[bibr34-11771801251363444] Monchalin R. Auger M. Jones C. Paul W. Loppie C. (2022). “I would just like to see more acknowledgement, respect and services for the people who are in between, just Métis people”: Recommendations by Métis women to improve access to health and social services in Victoria, Canada. AlterNative: An International Journal of Indigenous Peoples, 18(3), 327–334. 10.1177/11771801221103399

[bibr35-11771801251363444] Monchalin R. Monchalin L. (2018). Closing the health service gap: Métis women and solutions for culturally-safe health services. Journal of Indigenous Wellbeing, 3(1), 18–29.

[bibr36-11771801251363444] Monchalin R. Smylie J. Bourgeois C. (2020). “It’s not like I’m more Indigenous there and I’m less Indigenous here.”: Urban Métis women’s identity and access to health and social services in Toronto, Canada. AlterNative: An International Journal of Indigenous Peoples, 16(4), 323–331. 10.1177/1177180120967956

[bibr37-11771801251363444] Monchalin R. Smylie J. Bourgeois C. Firestone M. (2019). “I would prefer to have my health care provided over a cup of tea any day”: Recommendations by urban Métis women to improve access to health and social services in Toronto for the Métis community. AlterNative: An International Journal of Indigenous Peoples, 15(3), 217–225. 10.1177/1177180119866515

[bibr38-11771801251363444] Monchalin R. Smylie J. Nowgesic E. (2020). “I guess I shouldn’t come back here”: Racism and discrimination as a barrier to accessing health and social services for urban Métis women in Toronto, Canada. Journal of Racial and Ethnic Health Disparities, 7(2), 251–261. 10.1007/s40615-019-00653-1 31664676

[bibr39-11771801251363444] National Inquiry into Missing and Murdered Indigenous Women and Girls. (2019). Reclaiming power and place: The final report of the national inquiry into missing and murdered indigenous women and girls. https://www.mmiwg-ffada.ca/final-report/

[bibr40-11771801251363444] Paul W. Monchalin R. Auger M. Jones C. (2023). “By identifying myself as Métis, I didn’t feel safe. . .”: Experiences of navigating racism and discrimination among Métis women, two-spirit and gender diverse community members in Victoria, Canada. Journal of Health Services Research & Policy, 28(4), 244–251. 10.1177/13558196231188632 37436134 PMC10515468

[bibr41-11771801251363444] Payment D. (2001). “La Vie en Rose”? Métis Women at Batoche, 1870 to 1920. In Miller C. Churchryk P. (Eds.), Women of the First Nations (pp. 19–38). University of Manitoba Press.

[bibr42-11771801251363444] Payment D. (2009). The free people—Li gens libres: A history of the Métis community of Batoche, Saskatchewan. University of Calgary Press.

[bibr43-11771801251363444] Srugo S. A. Ricci C. Leason J. Jiang Y. Luo W. Nelson C. , & the Indigenous Advisory Committee. (2023). Disparities in primary and emergency health care among “off-reserve” Indigenous females compared with non-Indigenous females aged 15–55 years in Canada. Canadian Medical Association Journal, 195(33), E1097–E1111. 10.1503/cmaj.221407 PMC10462408 37640405

[bibr44-11771801251363444] Struck S. Enns J. E. Sanguins J. Chartier M. Nickel N. C. Chateau D. Sarkar J. Burland E. Hinds A. Katz A. Santos R. Chartrand A. F. Brownell M. (2021). An unconditional prenatal cash benefit is associated with improved birth and early childhood outcomes for Métis families in Manitoba, Canada. Children and Youth Services Review, 121, Article 105853. 10.1016/j.childyouth.2020.105853

[bibr45-11771801251363444] Teillet J. (2019). The Northwest is our mother: The story of Louis Riel’s people, the Métis Nation. HarperCollins Canada.

[bibr46-11771801251363444] Troupe C. L. (2009). Métis women: Social structure, urbanization and political activism, 1850–1980 [Master’s thesis, University of Saskatchewan]. HARVEST. https://harvest.usask.ca/handle/10388/etd-12112009-150223

[bibr47-11771801251363444] Truth and Reconciliation Commission of Canada. (2015). Truth and reconciliation commission of Canada: Calls to action. https://publications.gc.ca/site/eng/9.801236/publication.html

[bibr48-11771801251363444] Umaefulam V. Premkumar K. (2023). Enablers and barriers to diabetic retinopathy eye care among first nations and Métis women. Diabetic Medicine, 40(1), Article e14995. 10.1111/dme.14995 36308051

[bibr49-11771801251363444] Voaklander B. Sanni O. Serrano-Lomelin J. James A. Cordingley C. Bartel R. Eurich T. Ospina M. B. (2023). Diabetes during pregnancy among Métis people in Alberta: A retrospective cohort study. Canadian Medical Association Journal, 195(45), E1533–E1542. 10.1503/cmaj.230175 PMC10662493 37984935

[bibr50-11771801251363444] Wesche S. D. (2013). Métis women at risk: Health and service provision in urban British Columbia. Pimatisiwin: A Journal of Aboriginal & Indigenous Community Health, 11(2), 187–196.

